# Molecular characterization of the recombinant protein RmLTI-BmCG-LTB: Protective immunity against Rhipicephalus (Boophilus) microplus

**DOI:** 10.1371/journal.pone.0191596

**Published:** 2018-02-07

**Authors:** Bárbara Guimarães Csordas, Rodrigo Casquero Cunha, Marcos Valério Garcia, Sérgio Silva da Silva, Fábio Leivas Leite, Renato Andreotti

**Affiliations:** 1 Programa de Pós-graduação em Doenças Infecciosas e Parasitárias, Faculdade de Medicina, Universidade Federal de Mato Grosso do Sul, Bolsista de Doutorado pela Coordenação de Aperfeiçoamento de Pessoal de Nível Superior, Campo Grande, Mato Grosso do Sul, Brasil; 2 Programa de Pós-graduação em Biotecnologia, Universidade Federal de Pelotas, Pelotas, Rio Grande do Sul, Brasil; 3 Bolsista de Pós-Doutorado, Fundação de Apoio ao Desenvolvimento do Ensino, Ciência e Tecnologia do Estado de Mato Grosso do Sul, Campo Grande, Mato Grosso do Sul, Brasil; 4 Laboratório de Biologia Molecular do Carrapato, Departamento de Sanidade Animal, Embrapa Gado de Corte, Campo Grande, Mato Grosso do Sul, Brasil; 5 Laboratório de Doenças Parasitárias, Faculdade de Medicina Veterinária, Universidade Federal de Pelotas, Pelotas, Rio Grande do Sul, Brasil; Instituto Butantan, BRAZIL

## Abstract

The bovine tick *Rhipicephalus* (*Boophilus*) *microplus* is found in several tropical and subtropical regions of the world. This parasite transmits pathogens that cause disease, such as babesiosis (*Babesia bovis* and *B*. *bigemina*) and anaplasmosis (*Anaplasma marginale*). Tick infestations cause enormous livestock losses, and controlling tick infestations and the transmission of tick-borne diseases remains a challenge for the livestock industry. Because the currently available commercial vaccines offer only partial protection against *R*. (*B*.) *microplus*, there is a need for more efficient vaccines. Several recombinant antigens have been evaluated using different immunization strategies, and they show great promise. This work describes the construction and immunological characterization of a multi-antigen chimera composed of two *R*. (*B*.) *microplus* antigens (RmLTI and BmCG) and one *Escherichia coli* antigen (B subunit, LTB). The immunogenic regions of each antigen were selected and combined to encode a single polypeptide. The gene was cloned and expressed in *E*. *coli*. For all of the experiments, two groups (treated and control) of four Angus heifers (3–6 months old) were used. The inoculation was performed via intramuscular injection with 200 μg of purified recombinant chimeric protein and adjuvated. The chimeric protein was recognized by specific antibodies against each subunit and by sera from cattle inoculated with the chimera. Immunization of RmLTI-BmCG-LTB cattle reduced the number of adult female ticks by 6.29% and vaccination of cattle with the chimeric antigen provided 55.6% efficacy against *R*. (*B*.) *microplus* infestation. The results of this study indicate that the novel chimeric protein is a potential candidate for the future development of a more effective vaccine against *R*. (*B*.) *microplus*.

## Introduction

Vaccines containing the recombinant antigen Bm86 isolated from the *Rhipicephalus* (*Boophilus*) *microplus* intestine have been developed and marketed to induce immunological protection against tick infestation in cattle. Tick management through vaccination reduces environmental contamination and prevents the selection of drug-resistant ticks that results from the continuous application of acaricides. Tick vaccines are an attractive method for controlling ticks, but their efficacy needs to be improved. [1].

In Brazil, the vaccines TickGardPLUS (Intervet Australia) and Gavac^™^ (Heber Biotec S.A., Cuba) have lower efficiencies than vaccines from other countries (49.2% and 46.4%, respectively) [2]. Ideally, vaccine efficiency should be greater than 50%, as observed in studies involving recombinant proteins identified by sialotranscriptome analysis, where the vaccine conferred an efficacy of 73.2% [3].

To identify a new vaccine that can overcome the limitations of the currently available vaccines, a reverse vaccinology approach was used. Data generated by using the recombinant Bm86-Campo Grande antigen (BmCG) allowed the identification and characterization of recombinant antigens with immunogenic potential for use in more effective vaccines [4–6].

Researchers have used recombinant *R*. (*B*.) *microplus* antigens as protease inhibitors. These are part of a group of Kunitz protein molecules (BmTIs) that could potentially be used as immunogens against ticks. Some authors of this work previously reported the concentrations of BmTIs and found that they had increased specificity to neutrophil elastase during the egg to larvae phases [7]. These inhibitors may play a role in the feeding process of the larvae, and the use of antibodies against ticks may impair normal feeding and parasite viability [8]. The use of epitopes from Kunitz proteins in combination with immunogenic portions of other tick molecules in constructions based on multi-antigens represent an interesting strategy for combining proteins from one or more pathogens in a single molecule against *R*. (*B*.) *microplus*. This innovation provides a highly efficacious cattle tick vaccine [9].

The heat-labile enterotoxin B subunit from *Escherichia coli* (LTB) has been evaluated as a molecular adjuvant. This non-toxic subunit may have potent immunomodulatory activity as well as protective efficacy when fused or co-administered with a range of antigens. It is also considered a potent mucosal and parenteral adjuvant [10–13]. However, there are no reports of LTB being used in multi-antigen constructs against ticks.

The purpose of this study was to characterize the expression of a novel chimeric protein comprising parts of two LTB-fused proteins of *R*. (*B*.) *microplus* (rBmCG and rRmLTI) as a potential immunogen that can be used to control ectoparasites.

## Materials and methods

### *In silico* selection of coding sequences and gene design

The amino acid sequences of the proteins Bm86-CG (GenBank: ACA5782), BmTI (GenBank: P83606) and LTB (GenBank: ACJ23372) were used as references to design the chimeric gene (Table 1). These sequences were analyzed using the following bioinformatics software: SignalP 4.1 Server (http://www.cbs.dtu.dk/services/SignalP/), TMHMM Server v.2.0 (http://www.cbs.dtu.dk/services/TMHMM/), IEDB-Antibody Epitope Prediction (http://tools.iedb.org/bcell/) [14], *ProtScale* [15], and Vector NTI Advance^®^ 11 (Invitrogen) (www.invitrogen.com/VectorNTI).

**Table 1:** Amino acid sequences used to construct the recombinant protein.

Protein	Amino acids	Selected fragment (aa)
**Histidine**	HHHHHH	1–6
**RmLTI**	LEGSKRFETYCKPTHDRGPCKAYIPRWWFNVKTGQCEQFIYGGCQGNKNN	7–155
	YETKSICETNCLRRQLSELGVSADVHYRKHWNETKYSPNVTVEYPAVHFN	
	VTLNPVCNEP KYPELCKGYF PRYYYNSRSK TCKKFIYGGC QSNGNNFLT	
**BmCG**	RGRLRRSVCKAGVSCNENEQSECADKGQIFVYENGKANCQCPPDTKPGEIG	168–317
	CIERTTCNPKEIQECQDKKLECVYKNHKAECECPDDHECY	
**LTB**	HKMAPQTITELCSEYRNTQIYTINDKILSYTESMAGKREMVIITFKSGA	330–432
	TFQVEVPGSQHIDSQKKAIERMKDTLRITYLIETKIDKLCVWNQKTPNSIAAI	
**Linker**	SGGGGSGGGGS	(156–167), (318–329)

Regions that were surface-exposed, predominantly hydrophilic and contained a high number of linear epitopes were selected. Restriction sites for BamHI and HindIII were added flanking the gene, and flexible 2x SerGly linkers were inserted between the fragments to enable proper folding of the protein. The protein structure was predicted computationally by using the Swiss Model online server [16].

### Cloning, expression and purification of the recombinant protein

The rRmLTI-BmCG-LTB gene was chemically synthesized (GenOne, Brazil). The synthetic DNA was provided in the pAE vector [17]. The day prior to the transformation, the *E*. *coli* BL21 (DE3), C41 (DE3), and C43 (DE3) (CDTec-UFPEL) strains were streaked onto Luria-Bertani (LB) agar plates and incubated at 37°C. For transformation, 2 μL of plasmid (100 ng/μL) was added to a 1.5-mL microtube containing chemically competent cells [18], followed by application of the heat-shock protocol [19].

The isolated transformant colonies were inoculated into 250-mL Erlenmeyer flasks containing 75 mL of Terrific Broth (TB), 2x yeast extract-tryptone (2xYT) medium or Luria-Bertani broth (LB) culture medium [20] containing ampicillin (100 μg/ml) and incubated at 140 rpm for 16 h at 30°C.

Each pre-inoculum mixture was inoculated into two types of cultures at a 1:20 ratio. The first contained 500 mL of culture medium (TB, LB or 2x YT) in a 2-L Erlenmeyer flask, and addition of the pre-inoculum was followed by growth in an orbital shaker. The second type consisted of a 1.5-L stirred-tank bioreactor (B. Braun Biotech). In the latter case, 1 L of TB or 2x YT culture medium was used. For each fermenter in the bioreactor culture, the aeration rate was kept at 2.5 vvm, and the temperature was 30°C with stirring at 450 rpm. For the growth of the inoculum in the shaker, the culture was maintained at 180 rpm and 30°C. When necessary, foaming was controlled by adding anti-foamer (Silicone Antifoam-Sigma-Aldrich, SP, Brasil) at a final dilution of 1:1000 in both procedures. The expression of recombinant protein was performed using a previously described method [21], with some modifications. Recombinant protein was purified by using a HisTrap column (5 mL; GE Healthcare Life Science, Bio-Science Corp. Piscatway, USA) and the ÄKTA purification system (GE Healthcare Bio-Science Corp. Piscatway, USA) according to the manufacturer’s recommendations, with some modifications (10.17504/protocols.io.kiacuae).

### Cattle inoculations and challenge

The animals used were provided by a farm located in a rural zone (33 31′ 8″ S, 53 22′ 4″ W), Estância Três Marias (private property), Chui county, in the state of Rio Grande do Sul, Brazil. This location is considered a free zone of *R*. (*B*.) *microplus* infestation. For all of the experiments, two groups (treated and control) of four Angus heifers (3–6 months old) were used. The inoculation was performed via intramuscular injection with 200 μg of purified recombinant chimeric protein adjuvated with Montanide ISA 61 VG (Seppic) in a 60/40 (v/v) proportion of adjuvant/protein in a 2-mL volume per dose; the injections were performed three times at 15-day intervals [22]. The other four animals served as the control group and were treated per the protocol, except that they received 2-mL injections of adjuvant alone. Serum samples were taken from each animal before immunization and weekly thereafter. For the challenge, the animals were transferred to Hemotick Indústria e Comércio Ltda-ME, in São Lourenço do Sul- RS, Brazil. Twenty-one days after the last injection, the animals were challenged with 15,000 *R*. (*B*.) *microplus* larvae delivered along their back in three applications during the course of a week. Tick collections were performed daily once the engorged females started detaching spontaneously from the control animals. Tick samples were brought to the laboratory, weighed, and incubated at 29°C and 85% relative humidity until egg-laying was complete. Egg masses were weighed and incubated to determine hatch rate, i.e., fertility. All protocols were reviewed and approved by the Ethics Committee on Animal Experimentation (CEEA No. 4889–2015) of the Universidade Federal de Pelotas (UFPel). The CEEA of UFPel is accredited by the Brazilian National Council for Animal Experimentation Control (CONCEA).

### Antigenicity assays of the chimeric protein

To verify whether the antibodies induced by cattle immunization were able to recognize epitopes from rRmLTI-BmCG-LTB, we conducted western blotting and indirect ELISA using the method previously described [6], with some modifications (10.17504/protocols.io.kf8ctrw).

### Statistical analysis

Vaccination effects on tick biology and efficacy were determined as described previously [2, 23]. Briefly, reduction rates associated with rRmLTI-BmCG-LTB immunization relative to the unvaccinated group were determined for adult female ticks (% DT). Egg-laying capacity (% DO) and fertility (% DF) were also recorded. Vaccine efficacy was calculated as 100 × [1 –(CRT × CRW × CRO × CRF)]. CRT, CRO, CRW, and CRF are, respectively, the reduction in the number of adult female ticks, egg mean weight, egg-laying capacity, and fertility. The parametric *t*-test was used to compare biological data.

Mean antibody levels were determined for each group and compared using analysis of variance with 2 factors (ANOVA), and the F-test were used to determine the significance of any differences observed between the groups. Differences were considered significant at a P value < 0.01. The analyses were conducted using GraphPad Prism 1 Version 7 for Windows (La Jolla, USA).

## Results and discussion

### Chimera epitope prediction

Based on the bioinformatics analysis of the RmLTI, BmCG and LTB amino acid sequences, three portions with major epitopes were predicted. For a vaccine to be effective, it needs to promote and elicit a strong B cell and/or T cell response [24]. Due to the interactions between immune system cells and antigens in the development of a response, epitope mapping in the chimera is fundamentally important for choosing peptides with high vaccine potential [24].

**Fig 1:**
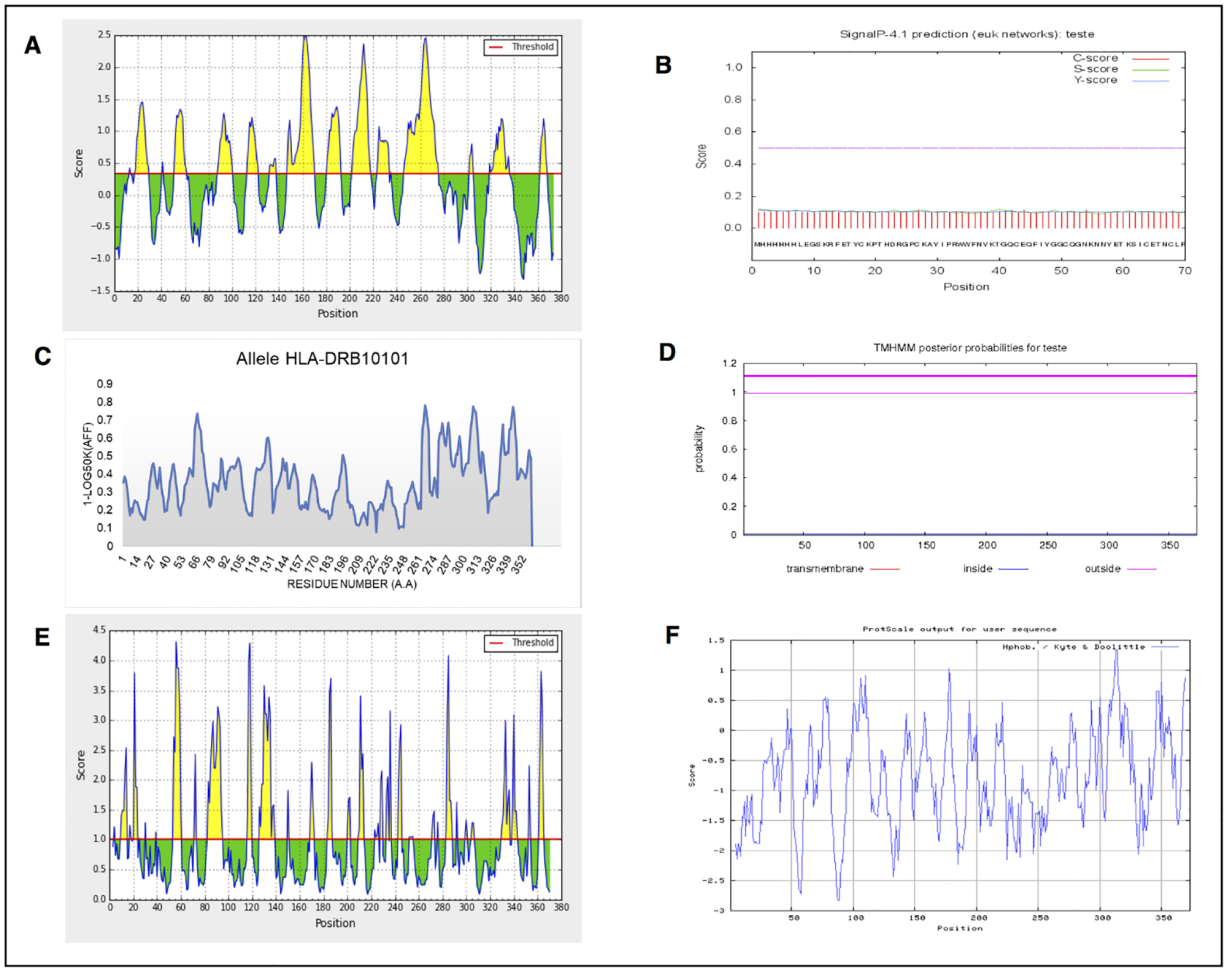
RmLTI-BmCG-LTB chimera analysis integrating different open-source algorithms. **A.** Prediction of linear B cell-binding epitopes **B.** SignalP prediction **C.** MHC-II (allele HLA-DRB10101) prediction. **D.** Transmembrane prediction (TMHMM). **E.** Surface exposure prediction. **F.** Hydrophilic or hydrophobic amino acid residue prediction.

The established criterion, which is based on the prediction of linear B cell-binding epitopes, aims to select sequences against which the host can trigger a humoral response in less time upon re-exposure to the antigen. Immunological memory is stimulated with boosters upon application of the chimeric antigens. The surface-exposed RmLTI-BmCG-LTB chimera tertiary structure regions were predicted to select the most exposed peptide sequences, to which the host-produced antibodies had a higher access probability (maximum score = 2490) (Fig 1A).

The signal peptide is cleaved post-translationally and is not retained in the final form of the protein [25]. Therefore, in the present study, the signal peptide sequences should not be included in the chimera because they are not normally present in the final structure of a mature protein. The chimeric signal peptide prediction does not contain this signal, as indicated by the score below the value indicative of a signal peptide (score < 0.2) (Fig 1B).

Regions with the potential to trigger cellular and humoral immune responses in humans were predicted because predictive tools for the MHCs of other species are insufficient, particularly with respect to class II receptor binding [26, 27]. In the present study, the prediction of the MHC-II allele (HLA-DRB10101) identified 26 strong bonds and 84 weak bonds (Fig 1C)

Transmembrane helix predictions were used to define signal peptide-containing regions, which should be avoided. Generally, these regions are located next to the lipid bilayer of the cell membrane, which hinders access by host antibodies [21]. In the present study, the transmembrane helix probability was low (> 1) (Fig 1D).

Prediction of surface-exposed chimeric regions was performed to select peptide sequences that were interesting targets because their tertiary structures were unstable and because they were located in a flexible region of the protein [28]. In the present study, we observed surface-exposed regions (Threshold = 1.0; score > 3.5) (Fig 1E).

A hydropathy index, which combines hydrophobicity and hydrophilicity, was used to predict which amino acids would be found in an aqueous environment (negative values) or in a hydrophobic environment (positive values). We observed higher peaks of hydrophobicity among the analyzed peptides using the *ProtScale* tool (Fig 1F) [15].

To construct a chimera that included immunogenic regions of both proteins, after selecting regions with linear epitopes, we ensured that complex post-transcriptional structures, such as glycosylation, did not interfere with the antigenicity of each protein. Because of the glycoprotein nature of these two tick proteins [4,7], we selected regions with linear epitopes, and we expressed the chimera in a system based on *E*. *coli*, which does not glycosylate proteins. Thus, glycosylation should not interfere with the linear structure of the antigen. If a eukaryotic expression system was used, such as *Pichia pastoris*, for example, the risk of these modifications interfering with the identity of the antigenic determinant would be high [5,19]. However, further studies must be performed to observe the behavior of glycosylation in this molecular structure.

### Vector construction

**Fig 2:**
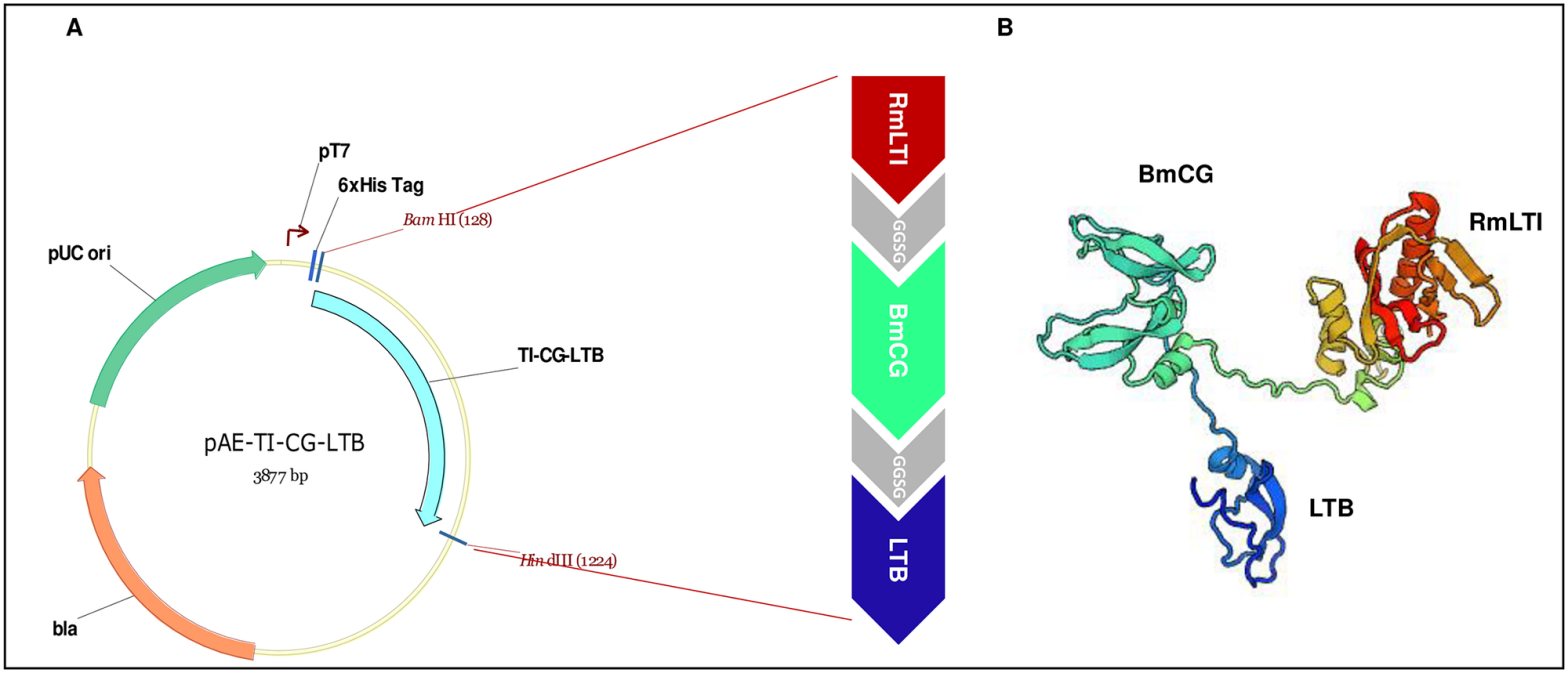
Schematic representation of the 3,877-bp plasmid encoding the chimeric protein. **A.** Expression cassette representation of pAE/RmTI-CG-LTB construction. The chimeric protein was fused at the 6xHis-tag of the pAE vector at the N-terminal region. Each chimeric subunit protein is connected by a 2x SerGly flexible linker, which is presented in gray. **B.** *In silico* prediction of the chimeric protein conformational structure.

The characteristics of the antigenic sequences used to build the chimeric protein are summarized in Table 1. The synthetic gene was cloned into the pAE expression vector (Fig 2A). The protein structure was predicted *in silico* using the I- Swiss Model online server [16], as shown in Fig 2B.

### Standardization of culture media in the shaker

**Fig 3:**
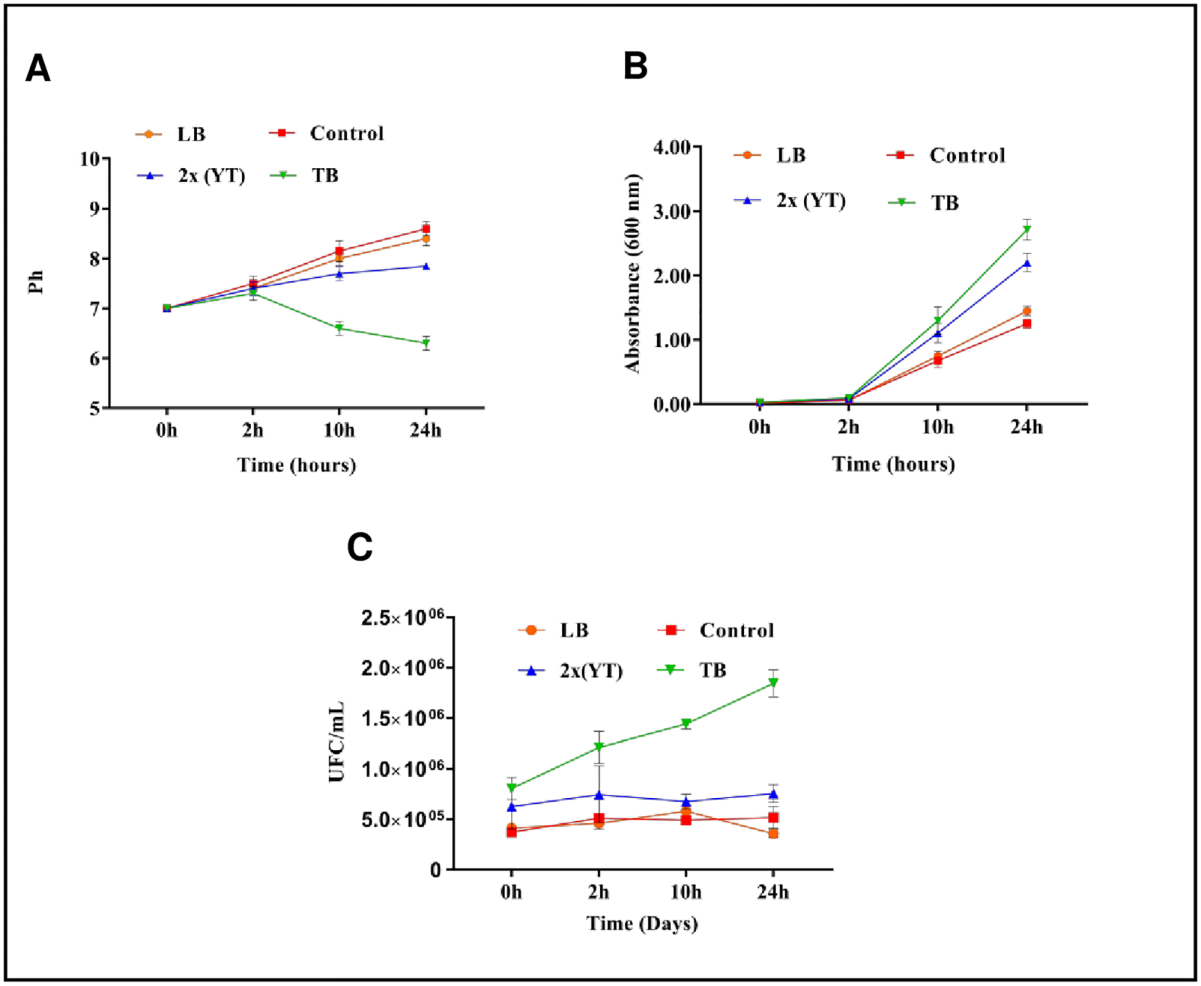
Comparison of different culture media at different time points. **A.** pH variation in the cell cultures in different culture media: LB, Control (*E*. *coli* strain C41 without the plasmid in LB culture medium), 2x (YT), and TB after induction. **B.** Variation in the absorbance after induction in the LB, Control, 2x (YT), and TB culture media. **C.** CFU/mL comparison among the LB, Control, 2x (YT), and TB culture media.

First, the culture media were standardized for production in shakers. Later, the media were standardized for production in a bioreactor. TB, 2x (YT), and LB culture media were considered, and 2x (YT) had a higher pH stability (Fig 3A), whereas LB had the lowest culture performance due to its alkaline pH value of 8.5. The level of acidity in the TB medium did not interfere with culture growth (CFU/mL) or with the OD600 absorbance readings (Fig 3B).

Aerobic growth of *E*. *coli* in the LB or 2x (YT) medium yielded an increasingly alkaline pH during the log phase due to amino acid metabolism, with further increases in the pH during the stationary phase [29]. This increase in the pH contributes to cell death and affects the onset of the stationary phase in the long term [30]. Therefore, it is important to end the induction period at 4 h to avoid cell death.

By comparing the cell viability (CFU/mL) of the expression strain studied, we obtained a peak of 2.0 × 10^6^ CFU/mL at 24 hours post-induction in the TB culture medium, followed by the LB and 2x (YT) media (approximately 1.0 × 10^6^ and 5.0 × 10^5^ CFU/mL, respectively) (Fig 3C). This increase in the OD600 absorbance in the TB medium compared to the 2x (YT) and LB media may be due to at least two factors: the presence of glycerol as a carbon source in the TB medium, whose metabolism releases an acid product into the environment, and the presence of potassium phosphate, which buffers this medium [31]. Therefore, due to alkalinity and low OD600, the LB culture medium was not considered for the bioreactor standardization studies.

### Bioreactor standardization and the RmLTI-BmCG-LTB chimera yield

**Fig 4:**
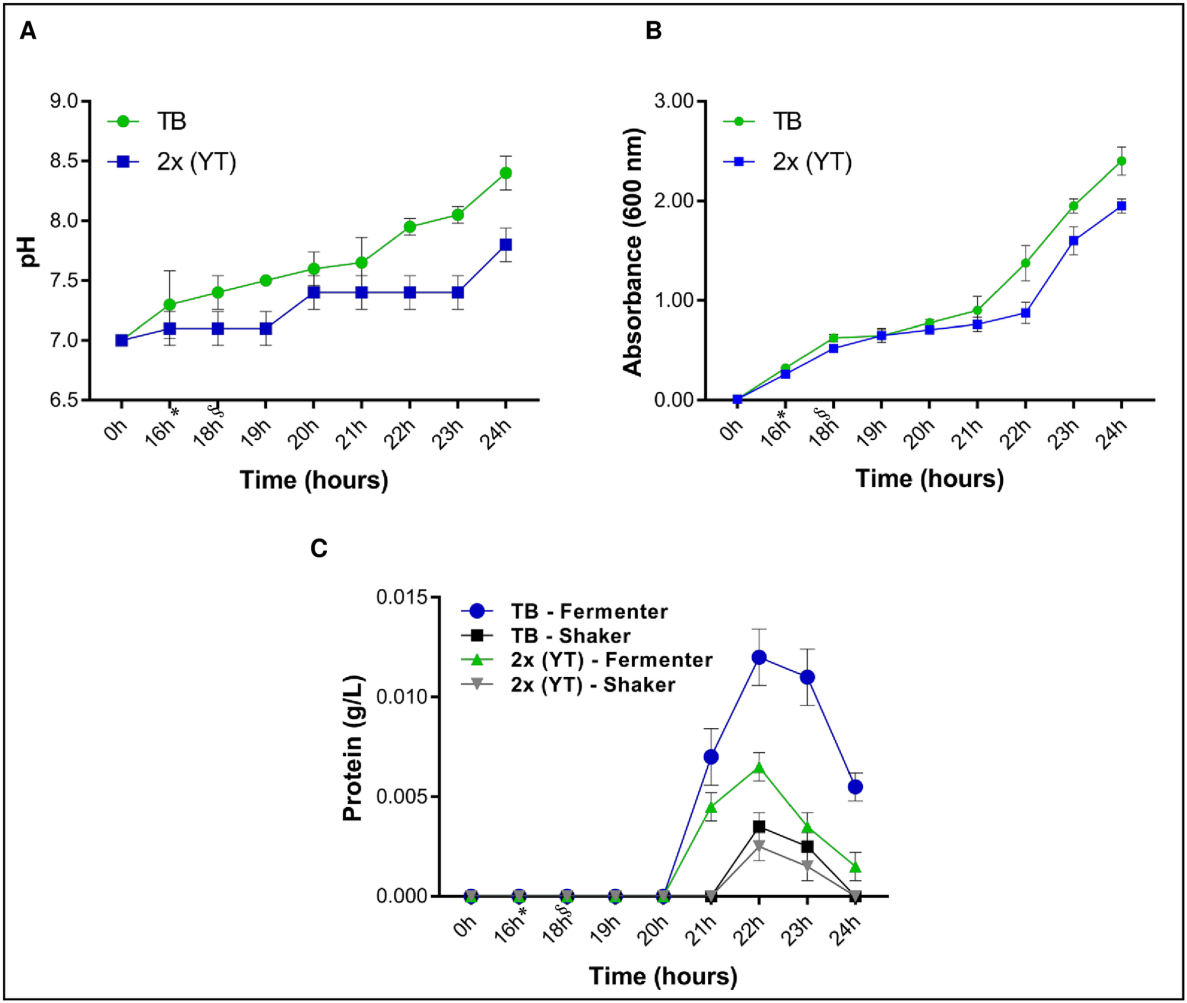
Bioreactor standardization and RmLTI-BmCG-LTB chimera yield. **A.** pH variation of the cultures in the TB and 2x (YT) culture media. **B.** Absorbance (A600 nm) variation of the TB and 2x (YT) culture media. **C.** TB culture medium in the fermenter (blue circle), TB and shaker (black square), 2x (YT) culture medium in the fermenter (green triangle) and shaker (inverted gray triangle). *Growth of the pre-inoculum in the fermenter. §Induction (1 mM IPTG).

By comparing the observed optical densities and the pH levels of the TB and 2x (YT) culture media, we found that the TB culture medium achieved the ideal OD for the induction (18 h) in a shorter period of time (OD = 0.6) than the 2x (YT) medium (OD = 0.5); however, the latter medium had a more stable pH under the same standardization conditions, which was similar to the results from the shakers (Fig 4A and 4B). At the end of the 24-h induction period, the TB culture medium had a higher OD (2.50 vs. 2.00). This OD increase remained in the bioreactor and shaker standardizations for TB, probably because the amount of carbohydrate supplied to the bacterium that was used as an expression system was 2x greater than the amount supplied by the 2x (YT) medium [32].

The culture medium and system with the best protein yield per volume was TB in the bioreactor (approximately 10 mg/L); the 2x (YT) medium in the bioreactor yielded almost half that value (5 mg/L of recombinant protein; Fig 4C). The higher growth yields observed in the fermenter can be ascribed to greater control of the aerobic culture conditions, which contributed more energy for protein synthesis [33].

The operation of the fermenter in discontinuous mode helps increase the culture biomass by ensuring that the oxygen supply does not become a limiting factor in the stimulation of acetate formation [34]. Another important factor to establish a good cell yield was maintaining the optimal growth temperature for *E*. *coli*. One study established that a range between 37°C and 39°C is optimal for the induction of the lac operon [35]. However, the use of lower temperatures reduces undesirable metabolic alterations, such as protease synthesis, which favor the yield of the product of interest and contribute to protein solubility, thereby reducing the amount of inclusion bodies [36]. Thus, the temperature used in this study was 30°C.

### Comparison of the elution fractions of the chimera in the shaker and fermenter

**Fig 5:**
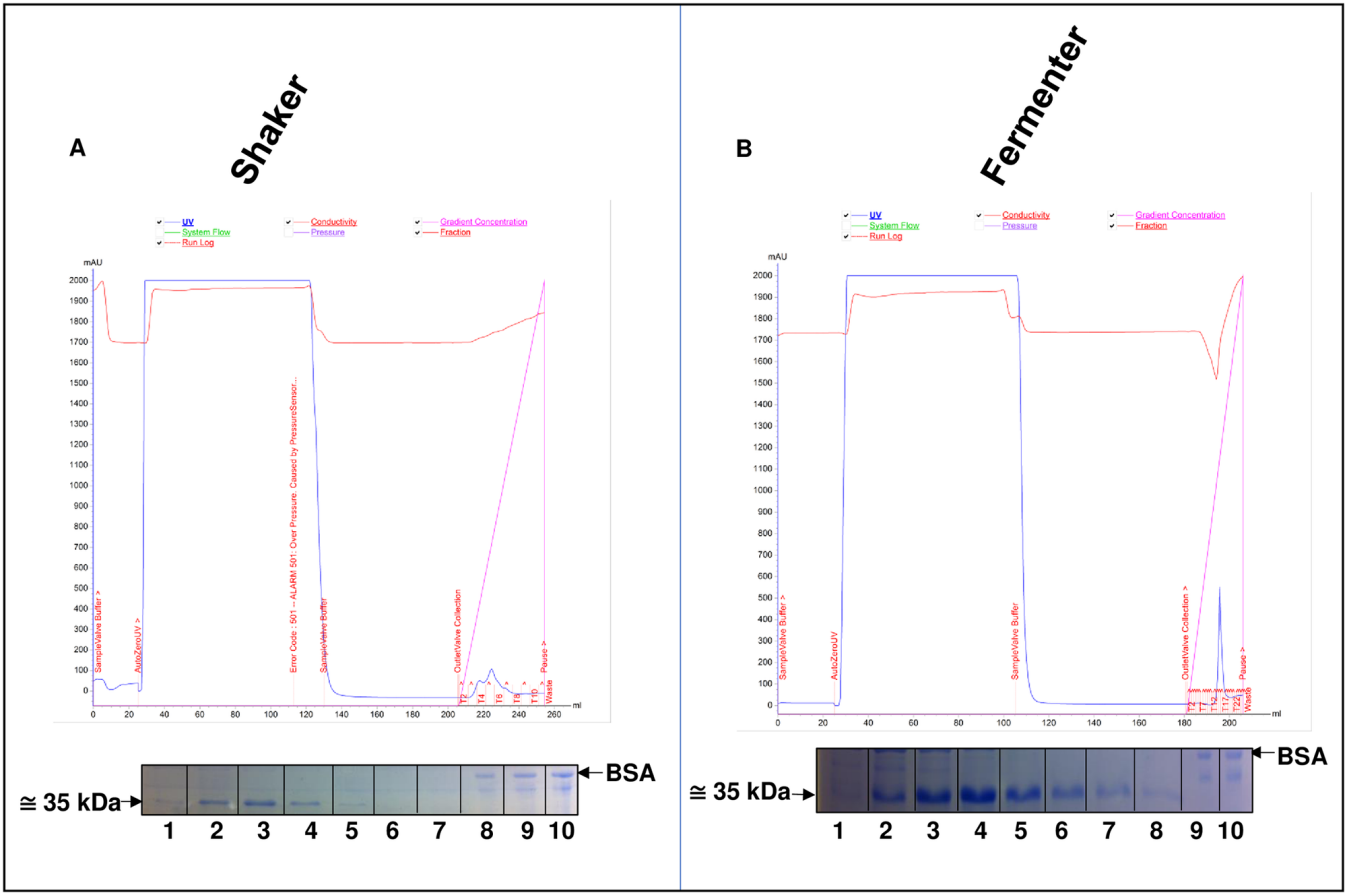
Purification of the recombinant protein using the ÄKTA purifier system (GE Healthcare Life Sciences). **A.** Purification of the protein expressed in the shaker. In the 10% polyacrylamide gel, wells 1 to 7 represent the imidazole-eluted fractions of the same recombinant protein. Well 8–0.5 μg of BSA. Well 9–1 μg of BSA. Well 10–2 μg of BSA. **B.** Purification of the protein expressed in the fermenter. In the 10% polyacrylamide gel, wells 1 to 8 represent the imidazole-eluted fractions of the recombinant protein (well 9 contains 1 μg of BSA, and well 10 contains 2 μg of BSA).

Elution of the recombinant protein expressed in the shaker system started at imidazole concentrations below 50 mM. However, the highest concentration of the chimera occurred in the fractions eluted with 50–200 mM imidazole (Fig 5A, wells 2, 3, and 4). Elution with imidazole concentrations above 200 mM resulted in low concentrations of recombinant protein (Fig 5A, well 5).

The recombinant protein expressed in the fermenter was not eluted at imidazole concentrations lower than 40 mM (Fig 5B, well 1). Elution of the recombinant protein was observed in SDS-PAGE with imidazole concentrations starting at 50 mM (Fig 5B, wells 2 to 8). A gradual decrease in elution occurred starting at imidazole concentrations of approx. 250 mM (Fig 5B, well 6, 7, and 8). Elution with 350 mM imidazole resulted in low concentrations of recombinant protein (Fig 5B, well 8). Therefore, elution of the protein was standardized using a buffer with a constant imidazole concentration set at 60 mM in a higher volume due to the high yield of this protein (approximately 10 mg/L) (Fig 4C).

To determine the amount of protein produced in the shaker and fermenter, the purified samples were subjected to 10% SDS-PAGE and compared with bovine serum albumin (BSA). The wells contained 0.5 to 1 μg of BSA (Fig 5A) and 1 to 2 μg of BSA (Fig 5B).

Denaturing solution was required due to protein insolubility, which was probably enhanced by the addition of LTB to the recombinant protein [17]. However, this issue did not impair the chimeric antigenicity observed in both the western blotting and ELISA assays.

### Immunoprotection

Daily collections of engorged ticks dropping from cattle were terminated 15 days after tick detachment commenced. The dynamics of engorged female detachment and egg production did not significantly affect the number of ticks. Egg mass and larval hatchability were also not significant. Immunization of RmLTI-BmCG-LTB cattle reduced the number of adult female ticks by 6.29%. There was no decrease in egg deposition capacity. Fertility was reduced by 19.25%, as shown in the Table 2. However, vaccination of cattle with the chimeric antigen provided 55.6% efficacy against *R*. (*B*.) *microplus* infestation.

**Table 2:** Effects on females and their progeny, and efficacy of vaccination with rRmTI-CG-LTB antigen against *R*. (*B*.) *microplus* that infest cattle.

Animal	Tick total number		Tick mean weight (mg)		Egg weight (mg)		Larval hatchability (%)	
	Control	Vaccinated	Control	Vaccinated	Control	Vaccinated	Control	Vaccinated
1	334	742	111,3	158,6	9,99	16,74	25,2	30,5
2	493	63	154,7	78,3	11,73	0,82	27,9	2,9
3	268	191	117,0	134,6	4,05	1,26	33,9	26,2
4	559	552	149,0	145,6	17,67	7,36	19,2	25,9
Mean ± SD^a^	413 ± 68	387 ± 157	133 ± 11,1	129,5 ± 17,8	10,86 ± 2,8	6,54 ± 3,71	26,5 ± 3,0	21,4 ± 6,2
t-Test	p = 0,8820		p = 0,8640		p = 0,3894		p = 0,4840	
% of reduction^b^	DT = 6,29		DW = 2,63		DO = 39,78		DF = 19,25	
% of efficacy =	100*[1-(387/413*129,5/133*6,54/10,86*21,4/26,5)] = 55,6%							

^a^Arithmetic mean ± standard deviation; p-values of t-test for independent samples are shown.

^b^Percent reduction was calculated in relation to the control unvaccinated group: DT, adult female ticks; DW, tick weight; DO, egg laying capacity; DF, fertility.

Efficacy% of efficacy = 100 [l − (CRT × CR0 × CRW × CRF)]; where CRT: reduction in the number of adult female ticks, CRO: reduction in the egg laying capacity, CRW: reduction in the egg mean weight, CRF: reduction in fertility.

Immunoprotection of cattle vaccinated with the experimental formulation rBm86-CG yielded a level of efficacy 18% lower than that reported with the Bm86-based vaccine Gavac^™^ against the *R*. (*B*.) *microplus* CG strain [2]. Studies with recombinant rBm86-CG [6] showed 31% efficacy in tick control. The recombinant protein rRmLTI showed an efficacy of 32% [7]. However, no study has shown whether there is any efficacy of the immunogenic potential or increased immune response against these proteins when they are fused.

The experimental vaccine formulation provided 55.6% efficacy, which could be due to the components that aided in the immunogenicity of rRmTI-CG-LTB. Advances in vaccinology have enabled the refinement of adjuvants and other components of antigen formulations. The water-in-oil adjuvant ISA 61 VG led to higher antibody titers compared to adjuvant ISA 201 VG and adjuvant Montanide Gel 01 [37]. The immunogenicity of the rRmTI-CG-LTB antigen could be enhanced by using other adjuvants. The vaccine with a mixture of various concentrations of Bm95 purified with aluminum hydroxide as an adjuvant protected the animals from the larval, nymph and adult tick challenges with efficiencies of 98.7%, 84.6% and 78.9%, respectively, in the control of *R*. *haemaphysaloides* infestations in the field [38].

### Chimeric protein antigenicity

**Fig 6:**
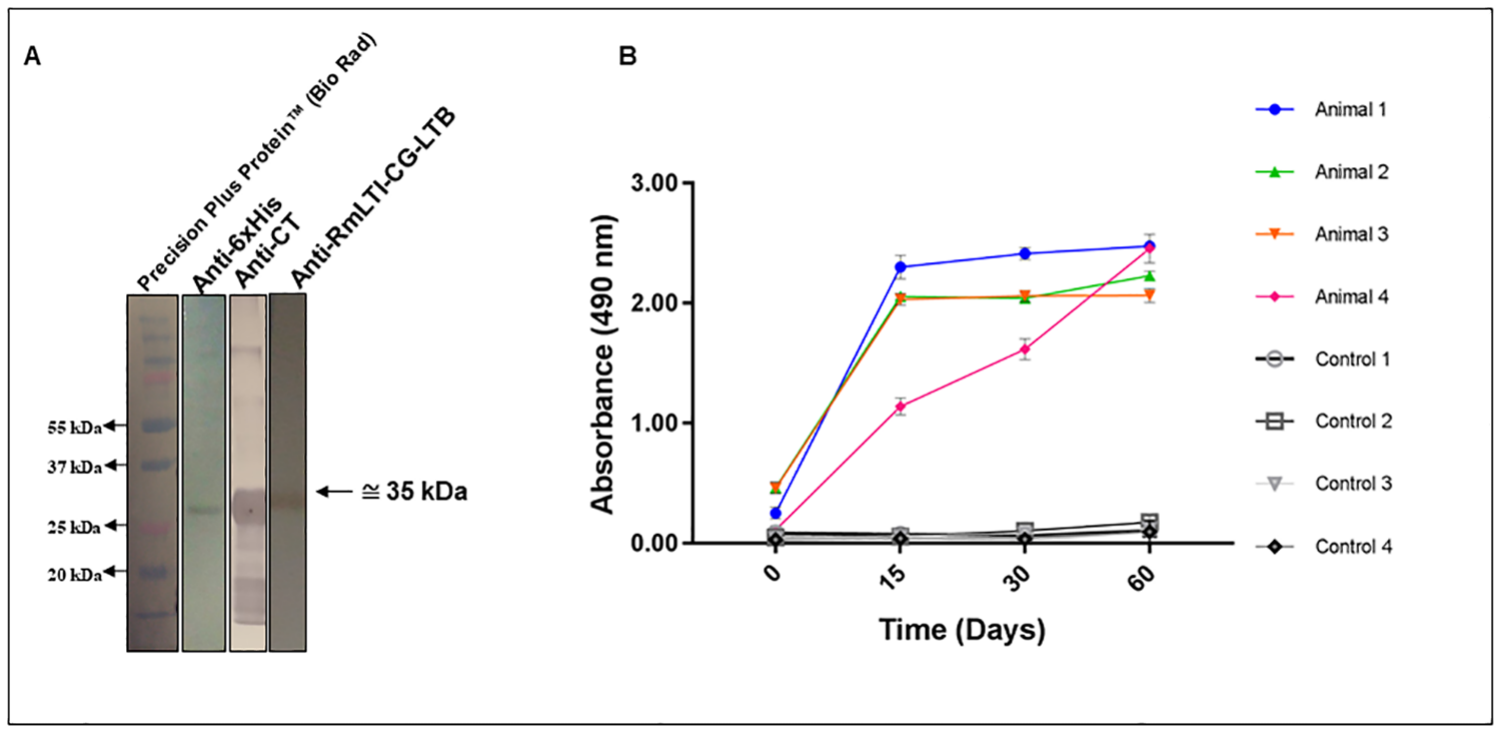
Antigenic analysis of the chimeric protein. **A.** Characterization by western blotting. The chimeric protein was specifically recognized by the anti-6xHis mAb (diluted 1:5000), the LTB mAb (anti-CT) (diluted 1:5000), and anti-RmLTI-BmCG-LTB bovine sera (diluted 1:50). **B.** Characterization by ELISA. The chimeric protein was tested against anti-RmLTI-BmCG-LTB bovine sera (diluted 1:800).

The western blot analysis of rRmLTI-BmCG-LTB revealed a band of approximately 35 kDa with affinity for the anti-His monoclonal antibody (mAb). The analysis also revealed recognition of the LTB portion by the anti-CT antibody and of the RmLTI-BmCG-LTB chimera by the sera from the vaccinated cows, indicating that this portion contained epitopes with antigenic and immunogenic potential (Fig 6A). Additional research involving the LTB protein demonstrated that this protein was essential for the development of a strong humoral immune response [24].

The portions of the recombinant protein corresponding to the RmLTI and Bm86-CG proteins in this study contained 269 of 373 a.a., including the linker-fused epitopes, the histidine tail, and the LTB (104 a.a.) (Table 1). The recombinant chimera had an estimated molecular weight of 42 kDa. Part of the LTB was dissociated, which was similar to reports of other chimeras [17]. Western blotting analysis using anti-CT polyclonal sera showed the presence of dissociated LTB (Fig 6A).

The antigenicity of rRmTI-CG-LTB was characterized by western blotting and indirect ELISA and was recognized by sera from cattle inoculated with the chimera (Fig 6B). Seroconversion of anti-rRmTI-CG-LTB was determined at the highest (1:800) dilution, with an OD492 nm > 2.8. Our results corroborate research on the characterization of the rBm86-CG protein [6] and the characterization of the rRmLTI portion [7].

The mean absorbance values for animals inoculated on the first day differ significantly (p <0.001) from the values obtained on days 15, 30, and 60 post-inoculation. However, the serum samples from days 15, 30, and 60 post-inoculation did not differ from one another. Thus, we can assume that the chimera is able to induce fast and effective antibody production by the immune system of the vaccinated animal and that the chimera is a promising candidate for vaccination against the bovine tick *R*. (*B*.) *microplus*.

## Conclusion

The expression of the recombinant RmTI-CG-LTB protein was optimized in TB culture medium with a yield of 10 mg/L^-1^ of medium. Additionally, antigenic determinants were retained in the protein, which showed promise as a protective immunogen. The high standard deviation presented in the number and weight of ticks are preliminary data that suggest protection (55.6%). However, this needs to be further evaluated in future studies.
